# Treatment by Vaginal Douches

**Published:** 1911-08

**Authors:** L. Sexton

**Affiliations:** Tulane


For Texas Medical Journal.

### Treatment by Vaginal Douches.

BY L. SEXTON, M. D., B. S., TULANE.

There is as much necessity and occasion for douching the vagina as there is for washing the mouth. Many patients who are scrupulously clean as regards the sanitation of the mouth seem to neglect or consider it immodest to use the ordinary cleansing douches which are so grateful, curative and helpful to the patient.

No treatment is more beneficial in vaginal diseases than the astringent or antiseptic douche at 110 degrees Fh. The larger the amount, and the warmer the temperature, the better the result. The nozzle of hard rubber, if wrapped in cloth, can be boiled and made aseptic just the same as the glass nozzle. It the douche is given to correct discharges it can be made astringent by adding acetate of lead, sulphate, zinc or pulverized alum, ten grains to the ounce. The vegetable astringents as tannic or gallic acid, may be used, as also hydrastis and hamamelis. Triodine two drams to the pint, or two per cent of permanganate are valuable antiseptic douches; the only objection to them being that they stain the clothes. All douches should be stopped the first two or three days of menstruation; as the period is coming to an end, douches are both cleansing and healthful. Two per cent of thymol is about the best deodorant douche we have in cancer cases. If the patient is wearing a pessary, the astringent douches adhere to and roughen its surface, so should not be used. The Murphy drip, or slow rectal douche, with saline or sterile water is a routine hospital treatment for all blood poisoning, hemorrhage and shocked cases, for uremia and for suppression of urine from any cause whatsoever. The quassia, or alkaline douche for the lower bowels in seat worms is an old tried and useful remedy. High rectal douches to unload the bowels and medicated douches for hemorrhoids are of proven value. They can be given to better advantage with the patient in the knee-elbow position. Where food can not be retained, as in stomach troubles, vomiting of pregnancy and in appendicitis, nutrient douches and enemas are used to advantage. In addition to the hot antiseptic douches in specific vaginitis or where the urethra is also affected, the latter should be washed out with a return flow catheter, or an application of nitrate of silver of the strength of ten grains to the ounce should be made by a pipette or applicator.

Fifty per cent of women in warm climates under forty-five years of age are troubled at some time with leucorrhoea. In the event this should have as a causative agent a run-down condition of the patient with a low red blood count, as a matter of course part of the treatment of such an anemic patient should be some tonic preparation containing iron, arsenic and strychnine, forced feeding and fresh air to build up the number of red blood corpuscles. The same anemic patient taking this constitutional treatment needs the local douching or the cure will not be complete.

Many other patients who are robust or plethoric suffer from leucorrhoea. In this condition the douching twice daily with some mild liquid antiseptic will relieve the case, provided the liver and bowels are stimulated to action by some suitable remedy at the same time.

Vaginitis specific, or non-specific, requires constant douching two or three times daily. In the specific variety, sublimate 1-5000 or 1-10,000, or a two per cent solution of nitrate of silver, two drams of tincture of iodine to the pint, or two per cent solution of boric acid, the latter being preferable if the inflammation and pain are severe. In using the douche under such circumstances it is better to fill the vagina entirely full of the solution so as to smooth out all wrinkles or sulci in order that the germicidal solution may come in contact with all disease germs. Any syringe which in a measure blocks the vaginal outlet while the canal is flooded with these antiseptic or germicidal solutions will do more good than one which simply runs the solution in and out without retaining it for any length of time. The common mistake in douches in the specific variety of vaginitis is using them too seldom instead of too often, and too strong instead of too weak. One-half of one per cent of carbolic solution, 1 to 10,000 bichloride, should be about the average strength used in specific vaginitis. Chancre and chancroids and the vaginitis which follow them call for the same solution and douching about which we have just spoken except the ulcers should be first cauterized with nitrate of silver or carbolic acid, which should be followed by douching with tincture of iodine, two drams to the pint, or the other antiseptic solutions alluded to before.

As said before, the vaginal douches should be at a temperature of 110 to 115 degrees Fh. if they can be borne that hot in order to get the best results. They should be given under pressure and continued for fifteen minutes at least. In subinvolution of the womb after labor or miscarriage, hot vaginal douches are of more virtue than ergot given internally; in such cases large quantities of very hot water are required for the douche. Intra-uterine douching after abortions or labor is only indicated when fever and chill produced by infection follows the absorption of decomposing material from inside the womb.

Hemorrhage from the womb may be relieved by hot astringent douches, Crede's compression, and the application of ice bags over the womb.

After parturition the morning and evening douches on the third day with a syringe, the nozzle of which does not extend far enough into the vagina for the stream of water to be forced into the womb, is a regulation order with many obstetricians and lying-in hospitals. Disagreeable odors and debris incident to parturition are removed by this douching. The comfort of the patient is much increased and convalescence accelerated by this simple treatment with any suitable antiseptic solution with which the doctor or midwife is familiar. There is no better way of relieving cystitis and urethritis than by large douches of mild antiseptic solutions of two per cent boric acid. These should be often repeated in order to accomplish the greatest good. All douches should be taken in the reclining position, preferably in a bath tub, in order to get the full benefit of the antiseptic and thorough washing out which is so essential in this method of treatment. The acrid discharges from the vagina often sets up a pruritus which is almost intolerable, except for the relief resulting from douches of carbolic acid and gum camphor in half dram to the ounce proportions, or only strong enough to produce a slight stinging; following this douching applications of five per cent carbolated vaseline, or dusting with any dry antiseptic powder or painting over the parts with a two per cent solution of cocaine often gives relief to a patient otherwise greatly inconvenienced. We are advocates of large families and against race suicide, and abortions at all times, but in cases of deformed pelvis and in some other conditions, it becomes absolutely necessary to prevent pregnancy rather than produce an abortion after conception has taken place. We can do this in no more simple way than by the use of bichloride tablets seven and one-half grains to the pint of hot water used as a douche immediately after any risk has been taken. If this is thoroughly and often repeated it reduces the chance of conception more than fifty per cent. Other germicidal lotions are perhaps as effective and less poisonous when the use of the bichloride tablets are feared, or not to be had. Not wishing to advertise any of the proprietary remedies put upon the market to be used as douches, we can state in general terms that any germicide which has proven its value and been endorsed by laboratory experiments, as germicides, may be used in vaginal douches, and the one with which the doctor is most familiar is the best for him to use in any given case. In ovarian inflammation, the ice bag externally, rest in bed, with hot antiseptic douches often averts an operation in the acute stage of the disease. The pre-operative douche, and scrubbing of the vagina is one of the greatest aids to a successful operation on the womb or vagina. This douching should be thorough and the antiseptic solution should be stronger than that used in inflammatory conditions of the vagina. As said before, all douches should be given in the recumbent position and as hot as can be borne, and with sufficient strength in the stream to wash away all vaginal discharges and decomposed material for which they are given. The more douches are used, the fewer pelvic operations required.

